# ‘The right not to know and the obligation to know’, response to commentaries

**DOI:** 10.1136/medethics-2020-106261

**Published:** 2020-04-29

**Authors:** Ben Davies

**Affiliations:** Uehiro Centre for Practical Ethics, University of Oxford, Oxford OX1 1PT, UK

**Keywords:** ethics

I am grateful for these four incisive commentaries on my paper, ‘The right not to know and the obligation to know’ and regret that I cannot address every point made in these challenging responses to my work.

Benjamin Berkman^1^ worries that I conflate medical information with medical action. I argue that patients sometimes have obligations to receive information, since medical decisions made with incomplete information may generate higher costs. As Berkman notes, though, information is no guarantee of action, and it is patients’ actions which will affect their health. Yet, he points out, I explicitly deny that patients should be ‘forced into a particular action’ (p.2). I acknowledge a missed opportunity to explicitly discuss the relationship between information and action. Still, Berkman’s discussion itself conflates two important ideas. As I argue, my having an obligation does not entail a permission for others to enforce that obligation. I do think patients sometimes have obligations to make certain health-related choices, specifically when this will not involve significant sacrifices of other values and will reduce future healthcare costs, but this does not mean anyone can legitimately *force* them to do so. Importantly, the obligation to be informed does not depend on your being in this position. If you face a reasonable chance of having a specific obligation, but do not know for sure, you ought to acquire the information needed to find out whether you are in an obligation-generating position. Berkman also notes that my argument does not consider whether the right not to know (RNTK) depends on explicit patient requests, or whether physicians should solicit patients’ consent before conveying information. Where possible, patients should be offered the opportunity to refuse information. But there are pragmatic limits; for instance, if an unexpected ailment is suspected, it is not possible to elicit patient preferences without giving them novel information.

Aisha Deslandes^2^ thinks the RNTK risks involving patients in the Sartrean idea of ‘bad faith’, offering the illusion of autonomy while forfeiting genuine freedom. On Deslandes’ view refusing relevant information involves denying our ‘subjective freedom’, which is akin to acting as if we were a ‘lifeless object’ (p.2), not an agent. This worry is significantly overstated. An individual can engage her practical agency in a wide range of ways while refusing medical information. Even if partial ignorance means a partial compromise of our agency, the idea that *any* refusal to embrace self-knowledge involves such a total objectification of ourselves is unjustified. Moreover, it is not clear that this really affects my argument. Deslandes proposes an ‘existential’ duty to confront knowledge, but unless others can legitimately *force* us to obey this duty—which seems inconsistent with a Sartrean view—this does not undermine the institutional right I propose.

Lisa Dive and Ainsley Newson^3^ think I presume an undefended liberalism, and that without such a perspective, ‘competing moral factors can be traded off against each other based on their merits, or the extent to which they contribute to a desirable end’. My most explicit invocation of liberalism is to say that *even with* a pluralistic liberal outlook, I believe that such an obligation is justified. But I am happy to go further: it is one thing to promote trading off moral factors by their contribution to a ‘desirable end’. But a liberal perspective will insist, rightly in my view, that it is the individual who must decide *which* ends are desirable. It is also unclear why liberals cannot trade competing moral factors ‘on their merits’. The paper’s definition of rights as non-absolute allows for this. But it does not mean that we should treat the RNTK as a mere preference. Dive and Newson suggest that I shift midway through my argument from discussing rights to discussing preferences, for example, in characterising the institutional RNTK as ‘a claim on health professionals…to respect a preference not to receive particular information’, (p.3) supporting their own view that the RNTK is merely a preference to be weighed against others. However, Dive and Newson have misunderstood my meaning: I do not anywhere characterise the RNTK as a preference. In the quoted excerpt, the word ‘claim’ stands in for right. Patients' preferences about their medical information give rise to claims on others, i.e. rights. However, this is not simply because they are preferences, but because they are preferences about a particular kind of information.

John Harris^4^ reiterates a common argument against autonomy-based defences of the RNTK, namely that since information is crucial for rational self-government, we cannot coherently defend ignorance in the name of autonomy. In my view, this claim contains an error parallel to that raised by Deslandes. No one can have perfect control over their life, or perfect information, and we must often make trade-offs. A decision to remain ignorant may reduce my autonomous control in some respects yet represent an expression of autonomy over others. Harris also suggests that a RNTK ignores the fact that ‘my right to tell the truth…however unwelcome to you, is at least as strong and often stronger than your claim not to hear them’ (p.2). As a moral claim, this relies on the ambiguity that I highlight in the paper: it may be true that I have a liberty-right to tell the truth; yet it may nonetheless be *wrong* for me to do so. Harris also presents a related argument that a legal RNTK worryingly constraints medical professionals’ free speech. Harris notes that truth is a ‘complete defence to charges of libel’. While this is true, it is somewhat selective. Truth is not always a defence, for instance, to breaches of confidentiality: my doctor cannot defend her decision to broadcast my medical details on the internet by noting that everything she says is true. Since both concern a patient’s control over their own medical data, confidentiality is in my view a more apt parallel than libel.

## References

[1] Berkman BE A commentary on ‘The right not to know and the obligation not to know’. J Med Ethics 2020;45:304–5. 10.1136/medethics-2020-106082 32350034

[2] Deslandes A Is the right not to know an instance of “bad faith”? J Med Ethics 2020;45:308. 10.1136/medethics-2020-106145 32350035

[3] Dive L, Newson A Obligations and preferences in knowing and not knowing: the importance of context. J Med Ethics;45:306–7. 10.1136/medethics-2020-106108 32350032

[4] Davies B The right not to know and the obligation to know. J Med Ethics. 2020;45:300–3. 10.1136/medethics-2019-106009 PMC7250654 32350031

